# Lethal perinatal hypophosphatasia caused by a novel compound heterozygous mutation: a case report

**DOI:** 10.1186/s12887-019-1478-7

**Published:** 2019-04-13

**Authors:** Fengdan Yu, Junyi Wang, Xiaojing Xu

**Affiliations:** Department of Neonatal Intensive Care Unit, The First Hospital of Tsinghua University, No. 6, Jiuxianqiao, Chaoyang District, Beijing, 100016 China

**Keywords:** Hypophosphatasia, Tissue non-specific alkaline phosphatase, Gene mutation

## Abstract

**Background:**

Hypophosphatasia (HPP) is a rare hereditary disorder characterized by defective bone and tooth mineralization and deficiency of tissue non-specific alkaline phosphatase (TNAP) activity. The clinical presentation of HPP is highly variable, and the prognosis for the infantile form is poor.

**Case presentation:**

This study reports a male infant diagnosed with lethal perinatal HPP. His gene analysis showed two heterozygous missense variants c.406C > T (p.R136C) and c.461C > T (p.A154V). The two mutations originated separately from his parents, consistent with autosomal recessive perinatal HPP, and the c.461C > T (p.A154V) was the novel mutation. Three-level structure model provide an explanation of the two mutated alleles correlating with the lethal phenotype of our patient. Results of SIFT, PolyPhen_2, and REVEL showed two mutations were pathogenic.

**Conclusions:**

We demonstrated a case of perinatal lethal HPP caused by two heterozygous mutations, and one of which was novel. This finding will prove relevant for genetic counseling and perinatal gene testing for affected families.

## Background

Hypophosphatasia (HPP) is a rare hereditary disorder characterized by defective bone and tooth mineralization and deficiency of tissue non-specific alkaline phosphatase (TNAP) activity [1], which was first described in 1948 by Rathbun [2]. The clinical presentation of HPP is highly variable, ranging from death in utero to adult dental problems and osteopenia. There are six subtypes of HPP including lethal perinatal, prenatal (or perinatal) benign, infantile, childhood, adult, and odontohypophosphatasia [3]. Lethal perinatal HPP is the most severe. Lethal perinatal and infantile forms are autosomal recessive, while the other milder forms are either autosomal dominant or recessive [3]. Babies affected with lethal perinatal HPP show rapidly worsening alterations of calcium/phosphate metabolism (hypercalcemia), apnea, seizures, and progressive encephalopathy. Severe respiratory problems, due to chest deformities and lung hypoplasia, are the direct cause of death. HPP affects all races around the world, with a highly variable prevalence. The prevalence of severe form is particularly high in American, Canada, European and Japan, estimated at 1:100,000, 1:100,000, 1:300,000 and 1:900, 000, respectively [4–8]. The clinical diagnosis of HPP is based on medical history, physical examination, laboratory findings, and typical X-ray skeletal alterations [9, 10]. In addition, genetic analysis is also an important form to clarify doubtful cases [3]. Analysis of the fetal DNA of cells obtained from the amniotic fluid has been used to diagnosis lethal perinatal HPP. Enzyme replacement therapy has been used to treat perinatal HPP in clinic [11].

In this study, we present a patient who was affected with lethal perinatal HPP because of a novel combination of heterozygous ALPL mutations. Two mutations, c.406C > T (p.R136C) and c.461C > T (p.A154V), originated separately from his parents, consistent with autosomal recessive perinatal HPP, and the c.461C > T (p.A154V) was the novel mutation. Three-dimensional structure model was used to predict functional impairment of the mutant TNAP protein, which provided an explanation of the two mutated alleles correlating with the lethal phenotype of our patient. The aim of our study was to improve the clinician’s understanding of the disease, strengthen genetic counseling and prenatal diagnosis, and reduce the birth rate of such children.

### Case presentation

A male infant was referred to our hospital due to tachypnea for 2 h after birth. He was a full-term infant of a G2P1 mother who had hypothyroidism and took euthyrox orally during pregnancy. His weight was 3560 g. Apger scores were 10 points and patient had no asphyxia after birth. Amniotic fluid was clear. Fetal heart monitoring suggested early deceleration, but there were no abnormality in umbilical cord and placenta. Prenatal B-scan ultrasonography at 25 weeks suggested that one side of the 2–4 vertebrae in fetal thoracic spine was small. However, complete fetal magnetic resonance imaging (MRI) showed no abnormality. Prenatal B-scan ultrasonography at 32 weeks suggested that the femurs were shorter than those at approximately 3 weeks gestation. The echoes on both sides of the thoracic spine were asymmetrical, and the corresponding parts of the spinal canal were thin. However, no more attention was paid to abnormal phenomena.

The infant gradually developed dyspnea 10 min after birth which was characterized by shortness of breath and cyanosis and accompanied by suction and sputum, and was then transferred to neonatal treatment. Physical examination results were as follows: his breath rate was 60 times / min, heart rate was 130 beats / min, length was 47 cm, head circumference was 34 cm, chest circumference was 31 cm. The symptoms of the patient were sobriety, poor response, convulsions, positive signs of three concaves, cyanosis of the lips. He had a short limbs, soft skull, narrow chest and soft abdomen. His bilateral lung breath sounds was rough without moist rale, heart sounds was strong and firm without pathologic murmur. His bowel sounds were normal, muscle force of the limbs was low, and the original reflection was incomplete. Blood test findings were as follows: PH 7.261, PO_2_ 38mmhg, PCO_2_ 55mmhg, Base excess 5 mmol/L, HCO_3_ 22.6 mmol/L, Haemachrome 18.4 g/dl, suggesting type II respiratory failure.

**Fig. 1:**
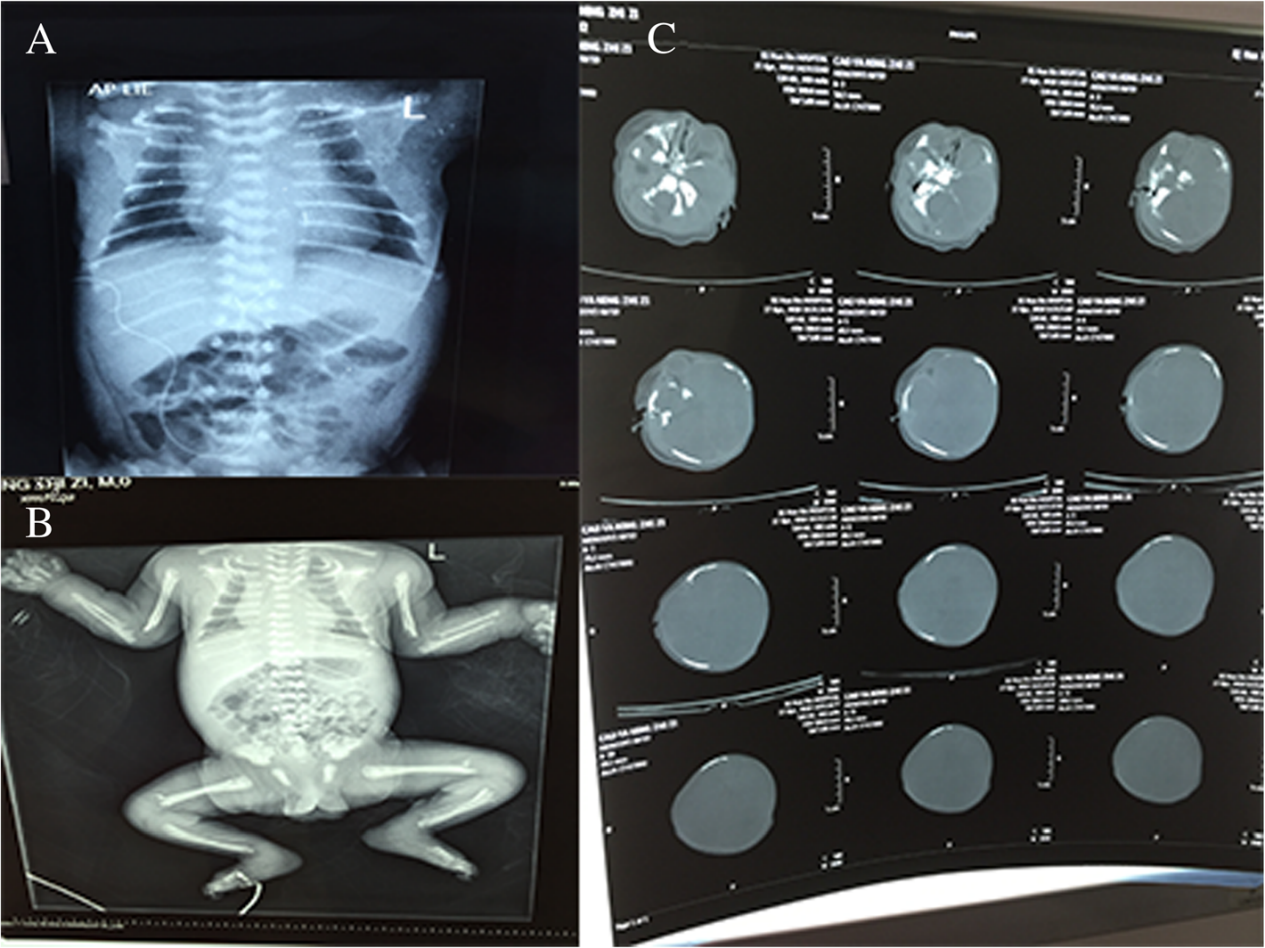
Patient radiography and CT. **a** thoracolumbar X-ray; (**b**), long bone of limbs x-ray; (c), patient Head CT

Non-invasive ventilator was given immediately after admission, the dyspnea was relieved, and blood gas returned to normal. However, the children suffered from recurrent dyspnea after withdrawal, which was aggravated after activities or crying. Oxygen delivery could not be stopped and needs to be used repeatedly because of the dynamic increase of partial pressure of CO_2_ in patients. On the 6th day after admission, epilepsies occurred, characterized by involuntary sucking movements, or systemic ankylosis, and the effect of anti-convulsant drugs was poor. Repeated dyspnea was a breakthrough point, the patient underwent chest X-ray, skull CT, long bone X-ray and laboratory examination. The chest and abdomen X-ray demonstrated thickened lung texture, visible ground-glass shadow, bell–shaped thoracic cage, thin ribs, and the absence of multiple attachments of the thoracolumbar spine (Fig. 1a). The X-ray of limb long bone demonstrated the bone characteristics on bilateral humerus, ulnar and radial bones, tibiofibula proximal and distal was irregular with multiple low-density lines. Bone fragments were seen on the distal femur (Fig. 1b). Head computed tomography (CT) demonstrated significantly reduced bone density and multiple skull osteogenesis imperfecta (Fig. 1c). Ophthalmologic consultation showed sclera was light blue. Serum biochemical test revealed that ALP was less than 5 IU/L in both measurements (normal range 45-125 IU/L). The level of blood calcium and phosphorus were normal. Based on the clinical and biochemical findings, the male infant was diagnosed as having HPP. Tracing the family history, his parents were asymptomatic, married and nonconsanguineous. To identify the underlying genetic defect, we performed molecular genetic testing for the ALPL gene. Parents were informed of the purpose of the study and signed the informed consent. The Ethics Committee of The First Hospital of Tsinghua University approved this study.

**Fig. 2:**
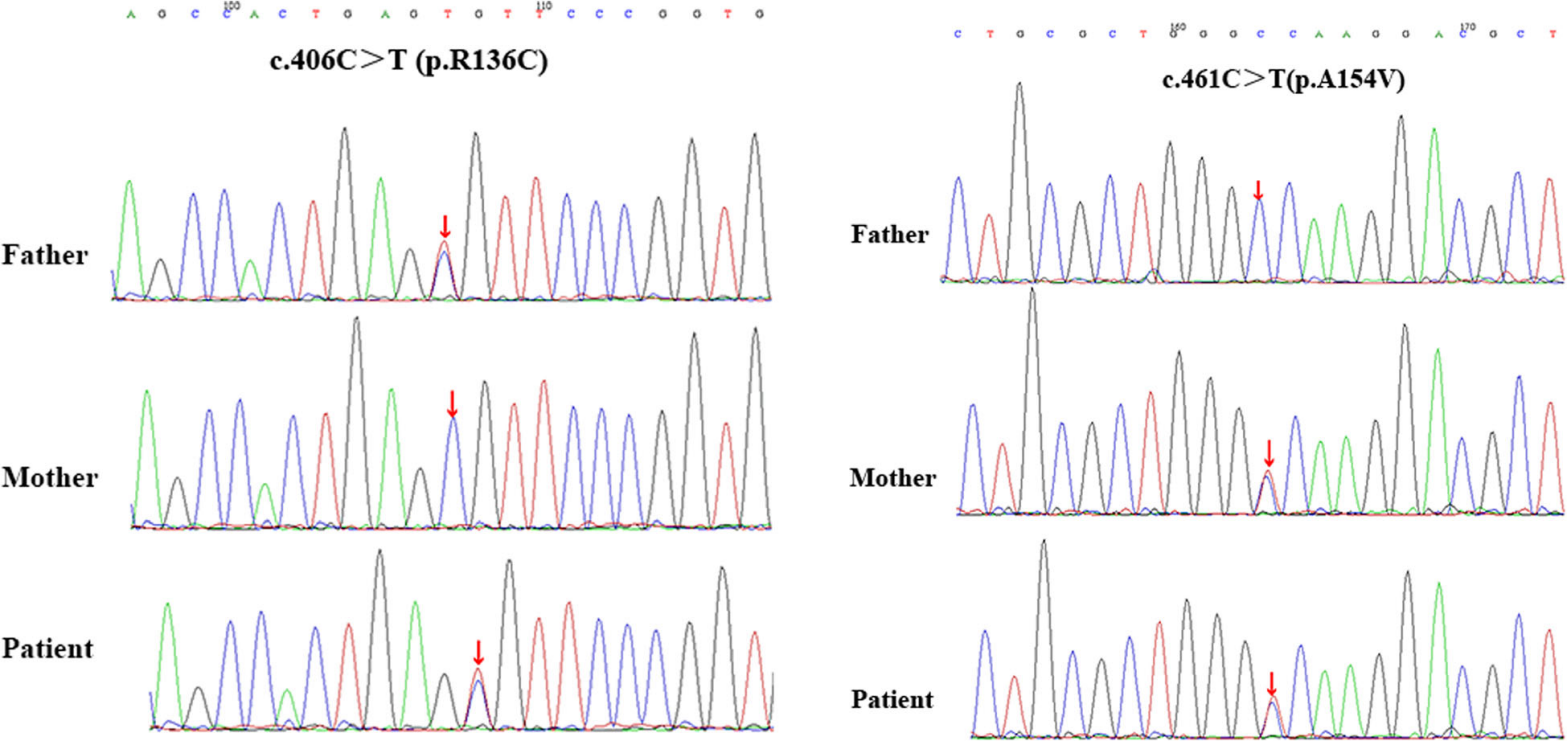
The sequencing results of the TNSALP gene in pedigree

Genomic DNA was isolated from peripheral blood leukocytes using the DNA purifcation kit (Omega Bio-tek, Inc., Norcross, USA) according to the manufacturer’s instructions. All coding exons and their flanking intronic sequences of the ALPL gene were amplified by polymerase chain reaction (PCR) using primers (Shanghai biological engineering co. LTD, Shanghai, China) on a thermal cycler (Biosystems, Foster City, CA, USA). Direct sequencing was performed using the same primer sets and ABIBigDye3.1 kit (Biosystems, Rotkreuz, Switzerland) on the ABI313OXI genetic analyzer (Biosystems, Foster City, CA, USA). To identify any sequence variants, the sequences were compared with reference sequences for the ALPL gene (GRCh37/hg19) using chromas sequencher software (Technelysium, Australia). Two heterozygous missense variants were found in both alleles of this patient; they were separately from his parents. The father’s mutation was c.406C > T (p.R136C) and mother’s mutation was c.461C > T (p.A154V). The father and mother of the infant were confirmed to be heterozygous carriers of each variant (Fig. 2). Genetic testing confirmed the diagnosis of HPP.

## Discussion and conclusions

The infant presented typical severe clinical manifestations, such as dyspnea, short limbs, respiratory failure, abnormal serum ALP, which were similar to previous report [3]. The patient gave up treatment for 19 days in hospital and died on the second day after discharge. His epilepsies did not improve after treatment with a variety of antiepileptic drugs. Epilepsy in infant HPP is usually associated with a deficiency of vitamin B6 in the central nervous system [12]. Pyridoxal 5′-phosphate, the active form of vitamin B6, involve in the synthesis of various neurotransmitters in the brain. Pyridoxal 5′-phosphate can be dephosphorylated by TNSALP. The defective metabolism in pyridoxal 5′-phosphate can lead to epilepsies [13]. Two mutations in the TNAP gene that resulted in the phenotype of lethal perinatal HPP were identified in this case. To our knowledge, the missense variant c.406C > T (p.R136C) has previously been reported [14], while the missense variant c.461C > T (p.A154V) was novel.

**Fig. 3:**
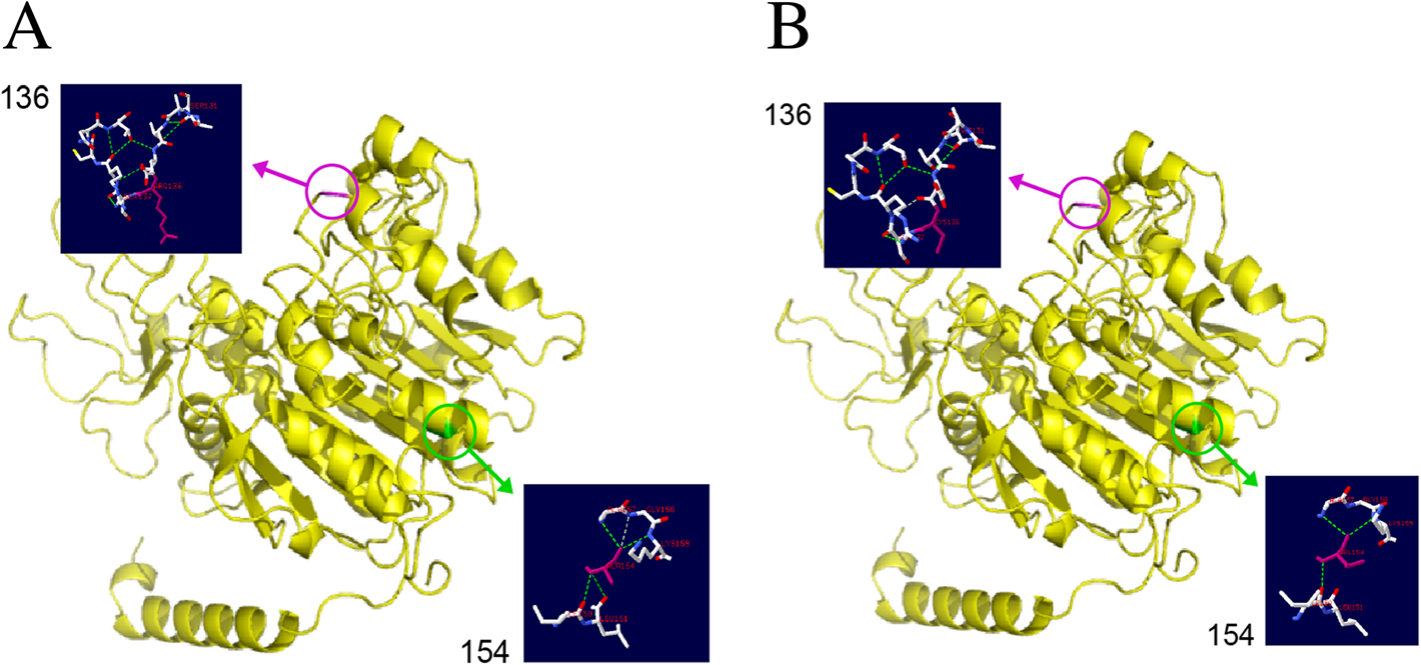
3D modeling structure of TNAP. **a** Ribbon presentation of the wild-type TNAP monomer. The purple circle represents the structure of 136 protein site in wild type; The green circle represents the structure of 154 protein site in wild type; (**b**) Ribbon presentation of the mutant-type TNAP monomer. The purple circle represents the structure of 136 protein site in mutant type; The green circle represents the structure of 154 protein site in mutant type

To investigate the correlation of phenotype and genotype, we analyzed protein functions using 3D structural analysis. It is necessary to analyze the association between genotypes and phenotypes to determine the role of each mutation in patient with compound heterozygosity of TNAP gene. Studies had shown that the mutation in gene can lead to various degrees of functional impairment and ultimately lead to the manifestation of various diseases [15–17].The Swiss-model online software (https://swissmodel.expasy.org/interactive) was used to construct the three-level structure model of wild-type and mutant TNAP protein. In the 3D structure, the mutation of c.406C > T led to the change of amino acids 136 from arginine to cysteine compared with the wild protein structure. The side chains of the amino acids were also changed after the mutation. However, the hydrogen bonds in the vicinity did not change. The mutation of c.461C > T led to the change of amino acids 154 from alanine to valine. The hydrogen bonds between 154 amino acid and 151 leu disappeared, and the hydrogen bonds between 154amino acid and 158 gly disappeared. The side chains of amino acids were also changed after the mutation (Fig. 3). In addition, three protein function prediction software SIFT, PolyPhen_2 and REVEL have shown that two missense variant in this study were pathogenic.

Up to now, there have been 388 genetic variations of the ALPL gene responsible for HPP (for a review, see ALPL gene mutations database on line: http://www.sesep.uvsq.fr/03_hypo_mutations.php). The clinical manifestations of HPP are highly variable, ranging from death in utero to adult dental problems and osteopenia. At present, enzyme replacement therapy has been used in clinic [11], and gene therapy is still under study. Genetic testing is used to diagnose hypophosphatemia. However, the results showed that the structure of these two mutants changed significantly and the damage of phosphatase function could be predicted well. These findings are related to the clinical presentation of the infant.

In conclusion, this study reported a rare case of perinatal HPP, which is caused by two heterozygous deleterious mutations (c.406C > T (p.R136C) and c.461C > T (p.A154V)) in the TNAP gene. Among them c.461C > T was a novel mutation. The results of 3D structural modeling showed that both mutations can led to significant structural alteration and the loss of phosphatase activity. Our study will promote the clinician’s understanding of the disease and strengthen the genetic counseling and prenatal diagnosis.

## Abbreviation

ALP: Alkaline phosphatase
CT: Computed tomography
HPP: Hypophosphatasia
MRI: Magnetic resonance imaging
TNAP: Tissue non-specific alkaline phosphatase
